# Emergency Remote Education, Family Support and the Digital Divide in the Context of the COVID-19 Lockdown

**DOI:** 10.3390/ijerph18157956

**Published:** 2021-07-28

**Authors:** María José Sosa Díaz

**Affiliations:** Department of Educational Sciences, Faculty of Teacher Training, University of Extremadura, 10003 Cáceres, Spain; mjosesosa@unex.es

**Keywords:** emergency remote education, online education, information and communication technologies, educational challenges, digital divide, social vulnerability, socio-digital inequalities, grounded theory, semi-structured interviews

## Abstract

To contain the COVID-19 pandemic, governments all over the world implemented strong lockdown measures to a large part of the population, including the closing of educational centres. Teachers were urged to transform their teaching methodology, moving from a face-to-face model to an emergency remote education (ERE) model, characterised by the use of technologies to continue with lectures and maintain the physical distance with the students. The aim of the present study was to analyse the existence of socio-digital inequalities and the educational challenges posed by the development of an ERE model, hence, contributing to the literature by proposing a systematic and holistic approach on this phenomenon. Based on the characteristics of the research problem and the objectives set, a qualitative methodology was applied. On the one hand, a semi-structured interview was conducted with 136 active teachers as the main data gathering technique. On the other hand, grounded theory was key in interpreting the results, with the aim of generating the theory in a systematic and holistic manner. It can be asserted that ERE was very useful during the lockdown of schools, and its potential to transform education was demonstrated. However, it was also shown that the development of an ERE model can cause socio-digital inequalities among students, due to the lack of access to digital devices and Internet connection, mainly due to factors, such as the socio-educational level of the family and the rural or urban context of the centre.

## 1. Introduction

In November 2019, a new coronavirus was discovered (SARS-CoV-2); a few months later, almost all of the countries in the world were affected by this disease. Every person is susceptible to the disease caused by SARS-CoV-2 (COVID-19), and in the absence of a vaccine or treatment to contain the pandemic, governments all over the world implemented strong lockdown measures to a large part of the population, including telecommuting and the closing of educational centres [1,2]. Kindergartens, schools, high schools and universities were closed. Approximately 1.6 billion students in 190 countries around the world stayed at home, thus, the pandemic affected 94% of the world’s school population [3]. The duration of the closing of educational centres ranged between 2 and 8 months, depending on the country [1]. In Spain, educational centres opened their doors after the holiday period, with a total of 6 months of inactivity.

In this context, teachers were urged to transform their teaching methodology, moving from a face-to-face model to an *emergency remote education* (ERE) model, in which they adapted their method to an online, virtual or remote teaching, depending on the circumstances of each education system, centre or teacher [4]. In this model, teachers use technologies as a key element to develop their lectures and maintain physical distance with the students [2,5,6,7,8,9,10,11,12,13,14,15].

Although there are differences in the way ERE is adapted or developed, common aspects can be identified. The aim is to favour a ‘ubiquitous’ learning, enabling its transferability to any space, context and time [8,16]. In this model, the teacher mainly aims to plan, organise and evaluate the contents, thus, the approach has shifted from teaching to learning, with a focus on the student [2,17]. In this sense, the use of didactic materials has increased, in order to transmit a message between the teacher and the students without physical contact [2]. Furthermore, the use of online resources has also increased, such as blogs and websites, as well as online collaborative tools and social network applications [13,14]. Videoconference and live recording were shown to be the predominant forms of communication between teachers, students and families [12,13,14].

It has been attempted to intentionally work on the autonomy of students, arouse their curiosity and motivation, and get them to acquire the new contents and relate them to the previous contents [2]. This is completed in order for them to learn action resources, while reducing the excessive dependence on the teacher and deriving a greater commitment toward learning [8].

However, it seems that the teaching model implemented by teachers depends on the model that was developed before the lockdown situation [8]. Therefore, those who use a competency-based model will carry out more active and applicable activities, whereas those who employ content-based models have maintained the unidirectional idea of sharing audio-visual documents or contents, instead of rethinking the approach toward more meaningful activities for the students [8].

The rapid transformation helped to maintain the continuity of the education programmes that are effectively adjusted for the purpose of completing the academic year [14]. Nevertheless, some studies state that the COVID-19 pandemic can be a constructive disruptor, as it has brought the opportunity to restructure the current classroom-based conventional education system [7,14,17]. Some researchers even point out that teachers have improved professionally, exploring new ways of teaching and the use of digital tools [17]. Some teachers have shown a great capacity to recover, demonstrating creativity and perseverance in responding to the challenging COVID-19 situation [18]. Thus, the closing of educational centres may have posed a chance to explore another education model, different learning options and other online tools [13].

However, this process did not occur without difficulties. The literature highlights that the lack of digital competencies among teachers [11,13], the attitude toward technologies and changed processes [15], the uncertainty of the time of the crisis and the family situation of each teacher have caused a high level of fatigue, stress and a negative emotional state during the transition to the new education model [10,15].

Furthermore, in an unprecedented situation, not only teachers had to adapt immediately to a digital education environment, but also the entire educational community [2,10,18]. In most cases, this was without a previous plan and not always with the adequate resources [11,16]. Firstly, students face a challenge characterised by the predominating use of online learning management systems and technological applications [6,7]. Secondly, families had to help their children with the virtual homework [8,11,13,15]. Lastly, the educational institutions had to improvise with the available technological means, to allow and help students to continue with their learning [5,8].

Numerous studies have found that adapting to the new model is not easy, due to the difficult access to and availability of technological devices and the Internet among students, for the adequate development of learning [7,10,11,12,13]. Suddenly, in the process of change and transformation toward online teaching methods, the digital inequalities among students are revealed [6,13].

During the last two decades, ICTs have posed a radical change in society, and generated a social phenomenon of inequality as a result of their rapid and continuous technological development in every human activity [19]. As a complex and multi-level phenomenon, they have captured the interest of international research since the late 20th century, and they continue to be a central issue of thematic discussions and debates in different studies [19,20,21].

The understanding behind most of the research in this topic is that the digital divide is a complex issue, and it is difficult to comprehend the phenomenon within a single context and with a single definition. Initially, the conceptualisation of the digital divide was developed from a dichotomous category: access or no access to technology [22]. However, in reality, the phenomenon is much more complex, due to a variety of economic, demographic, cultural, individual and social factors [22,23,24,25]. Moreover, these factors are often interdependent; this gave rise to the term ‘intersectionality’, which is focused on individuals who are affected by multiple inequalities [21,22,26]. As in the case of poverty, digital exclusion is an integral part of a social structure and processes, and it is not easily understood by researchers [24].

The digital divide is defined as a social inequality between individuals regarding (1) “access” (availability of technological devices and Internet connection), (2) “effective use” (frequency of technology use) and (3) “social envelope” (capacity to use ICT for different purposes) [20,25,27]. Thus, there are three types of digital divides, based on their nature and associated factors: (a) access to technology, (b) digital competency and (c) attitudes and motivation toward technologies [23]. The three categories of digital divides are not independent from each other [20]. The interaction among them is the foundation on which learning technologies pave the road to the academic and social development of students [23]; therefore, the digital divide can be related to academic performance in general [28].

Although it seemed that the problem of the digital divide was solved, recent studies have explored factors that influence the digital divide, such as gender [28], socio-economic and cultural level [23,28,29,30], functional diversity [31], the rural/urban context [32,33] and race and ethnicity [26]. All of these factors confirm that before the pandemic situation, the digital divide still represented a great social challenge, revealing that educational centres had to develop effective strategies to balance the social and learning opportunities among students [32].

The technological divide has become more evident than ever since the closing of educational centres due to the COVID-19 pandemic [16]. Numerous studies have analysed the digital divide in the educational scope as a result of this pandemic [5,6,8,11,15,17,18,29,34,35]. The results show important barriers to the learning and effective participation of students, in conditions of equity and equal opportunities [11,18].

The findings show that digital inequalities can increase the vulnerability of groups that are already at risk [26]. It has been pointed out that students of lower socio-economic levels are disproportionately affected by the pandemic, mainly due to a lack of access to technological devices [5,10,13,17,18,29,36]; the lack of previous training and digital competencies of the students [6,13,18]; and the absence of other enriching opportunities at home [36]. Moreover, some studies have found significant differences in the access to digital tools and remote learning opportunities during the pandemic between students from public schools and those from private schools [29]. Furthermore, other studies report the existence of inequality between students from rural schools and those from urban schools [5,6,34], since the digital disconnect is much greater in the rural scope [5,12,34].

However, the digital divide goes beyond the availability of technological devices; despite having basic materials, many students have had great difficulties in continuing with the academic course and could not attain the minimum objectives in this lockdown period [5,11]. In this context, some studies confirm that the students that are most vulnerable to the digital divide, such as students with special educational needs and/or functional diversity and those from ethnic minorities, have fewer learning opportunities, and the ones that they have are worsened by the COVID-19 situation [11,17,37].

Other studies highlight that students from families with low levels of commitment and participation [18,34,36], and reduced capacity to support the remote education of children during the pandemic are also in a situation of social inequality and inequity [29,38]. Children depend largely on the participation of their parents in their basic education [18]. In this context, the academic results during the closing of educational centres have become more dependent on motivation, attention, attitude, parental skills and the accompaniment strategies of the families [35,39]. Moreover, the low computing skills of parents in the learning environment at home [25] and the poor academic and digital literacy in the family environment [8,16,36] can worsen the socio-digital inequalities among students.

Therefore, the inequalities among students can be strongly affected by the economic/social/cultural/digital environment at home. The variables of the context, related to the family situation of students (e.g., economic situation, labour demands, availability of and access to digital devices, and the educational style), constitute first-order aspects to work on during lockdown periods [11].

Following the international interest of the digital divide and the educational change during the lockdown period, the aim of the present study was to analyse the existence of socio-digital inequalities and the educational challenges caused by the development of an *emergency remote education* (ERE) model. This study contributes to the literature by proposing a systematic and holistic approach on this phenomenon. For the attainment of such an objective, it was necessary to establish research questions and design the process that would be carried out. The questions posed were extracted from a thorough literature review on the topics of interest, and they make up the conceptual structure for the researcher, in order to focus on the important aspects of the study [40].

(a) What methodological changes were made to adapt to ERE?
(b) What challenges did teachers and the educational community face during the lockdown?
(c) How did the socio-digital inequalities manifest throughout the lockdown?
(d) How did families address their participation and accompaniment in the learning of their children?

## 2. Materials and Methods

### 2.1. Research Methodology

Based on the characteristics of the specific research problem and the objectives set, a qualitative methodology was selected for different reasons. Firstly, qualitative research implies an interpretative and naturalistic approach to the world [40], which allows the study of things in their natural context, aiming to make sense of or interpret phenomena according to the meaning that people give them [41]. Moreover, the digital divide and the process of educational transformation are complex phenomena, and only qualitative methodologies allow understanding and interpretation of this type of phenomena from a comprehensive, holistic and profound perspective [42]. In this sense, the search for causal relationships is disregarded, focusing on the establishment of global relationships, and ensuring the preservation of the complexity of the phenomena and the direct applicability in the context.

### 2.2. Data Collection Techniques

For the attainment of the research objective and questions, a semi-structured interview was used, along with a script of questions, as the main data collection technique. An interview can be understood as a dialogue initiated by the interviewer with the specific purpose of obtaining relevant information for the development of an investigation [43]. In this sense, it provides the perspectives of the interviewees on the analysed phenomenon in detail. The interviews were conducted in two phases:

(a) Compilation of data/characteristics of the sample through Google Forms, with the aim of recruiting participants for the study.

(b) Realisation of individual interviews through videoconference, with the aim of gathering the relevant data and recording the conversation.

The interviews employed in the present study were semi-structured, and based on pre-formulated dimensions with a pre-established order, although these could vary during the conversation. Thus, the protocol of the interviews (see Appendix A) had a pre-determined structured, which the researcher prepared before the meeting.

### 2.3. Participants

The population of informants includes every person related to the study object who can provide relevant information about the analysed phenomenon [44]. Clearly, not all informants could be selected; thus, for the present study, active teachers who would be willing to participate were previously reached through a Google Form. Those who replied to the questionnaire were invited to participate in the interview through a videoconference. To control for the characteristics of the interviewed sample, the questionnaire included questions about different context aspects, which are described in detail below.

- *Number of participants*: a total of 136 active teachers were interviewed.
- *Autonomous community to which the teachers belonged*: The participants belonged to four main autonomous communities. Of the total sample, 38.2% belonged to educational centres of Extremadura, 39.2% belonged to the community of Madrid, 12.8% to Castile La Mancha and 9.8% to the community of Valencia.
- *Educational stage*: in total, 33.08% of the participants taught in early childhood education, 41.17% in primary education, 23.52% in secondary education and 2.20% work with students with special educational needs or needs in hearing and speech.
- *Socio-cultural level of the area of the educational centre*: 61.8% of the participants belonged to a medium socio-cultural level, 20.6% to a high socio-cultural level and 17.6% to a low socio-cultural level.
- *Types of educational centres of the participants*: 76.5% were public centres and 23.5% were charter centres. None of the participants taught in a private centre.
- *Educational context of the educational centres*: 73.5% were in an urban context and 26.5% were in a rural context.

### 2.4. Data Analysis

Grounded theory (a comparative method) was essential for the interpretation of the results [42,45]. The constant comparison method from grounded theory was adapted to the objectives of the present study. This method consists of the search for similarities and differences through the analysis of incidents contained in the data, through the simultaneous processes of coding and analysis, with the aim of generating a theory systematically [46]. This allowed the identification of four main stages from the analysis of the data:

*Stage 1—open coding*. This is defined as “the analytical process through which the concepts are identified and the properties and dimensions of the data are discovered” [42]. Therefore, the first step was to categorise and compare the data, considering the system of categories and dimensions that emerged from the data (Table 1). Thus, a microanalysis of the information was performed, dividing it into data subunits and assigning the data to subunits that were significant for the different categories and dimensions.

*Stage 2—axial coding.* This procedure is defined as a thorough analysis of a category, aimed at identifying the interactions and relationships between such a category and other categories/subcategories or properties [42]. This analysis of data was performed along with the physical grouping of each of the information subunits that were interpreted to belong to the same category. The creation of matrices has become a very useful method for the formulation and validation of conjectures [47]. In this procedure, we identified the most common data and which participants they came from, allowing us to consider, reflect on and determine the main ideas, as well as to answer questions that typical of axial coding, such as *why, where, when and with what does this occur?* [42]. Lastly, we carried out a synthesis and selection of the most important information, and created memorandums, in which the first impressions of the gathered data were specified.

*Stage 3—selective or theoretical coding.* Theoretical codes “weave the fractured story back together again” [48]. At this point, the data were recomposed to make sense of the emergent analysis. Thus, using the matrices, memorandums and the most relevant information, the theoretical writing was carried out, specifying the findings, integrating results and building conceptual maps defined by [42] as *“graphic representations or visual images of the relationships between concepts”.*

*Stage 4—theoretical saturation.* The data gathering was terminated, as no more new ideas or relationships appeared, proceeding to the description of the results [42].

### 2.5. Analysis Instruments: Memorandums

According to [42], memorandums (or “memos”) are notes written by the researcher during analysis, in order to remember some aspects or as a source of information. The production of memos is a constant process [46]. They gather the thoughts and interpretations about data, the explanations of the concepts and categories and also the directions that the analysis should follow [49].

Each of the memorandums shows the entire evolutionary process of the constant comparison method of a category, and, therefore, of the different coding procedures (open, axial and selective). All the memorandums have the following parts:

1. *Analysis of the content of the sources:* this section shows the interpretation of each of the categories classified by codes.
2. *Matrices and explanatory tables:* at this point, a table was organised with the ideas that appeared in the microanalysis, indicating the number of times that a specific idea was mentioned.
3. *Main ideas:* the summary and selection of the most important ideas, and a description of the relationships between the found concepts.
4. *Conceptual map:* the relationships found between concepts are visually represented.
5. *Questions:* a set of questions that guided the research were proposed, and it was indicated whether the categories seemed saturated or not.

## 3. Results

### 3.1. Educational Change during the Lockdown

#### 3.1.1. Key Elements in ERE during the Lockdown

The methodology and activities used by the participants during the lockdown were diverse, although, in general, they highlighted the passive use of digital didactic resources. Most of the teachers, especially those in higher educational stages (primary education and secondary education), adapted the traditional methodology of content transfer (76 participants) mainly asynchronically (42 participants), since *“the methodology was based on videos that existed already or videos created by the teachers with their own explanations, followed by activities from the textbooks, enhancement activities in the classroom, evaluation forms, etc.”* (p111, MET_ACT_ESAL). Thus, students and their families could access the content from any device at any time (8 participants). However, other teachers chose ordinary lectures, similar to the ones they carry out in their classroom, delivered through videoconference and supported by other resources, such as textbook activities (34 participants).

*“During the state of alert, we adapted the class to teach the same content through videocalls, and, for those students who did not have digital devices, the activities and explanations were sent via ordinary mail. (p75, MET_ACT_ESAL)”.*

Some of the participants, especially those who taught younger students (early childhood education), preferred more active educational models to facilitate their learning and make it more motivating. For example, using more didactive and manipulative materials, such as interactive videos, educational online games, musical videos, etc. (31 participants).

*“Well, I used games, manipulative activities, videos, interactive books and forms (p122, MER_ACT_ESAL)”.*

A lower number of participants also carried out more active emergent methodologies with their students, through developing cooperative methodologies, project-based learning, design thinking and flipped classroom (25 participants). Through these didactic methodologies, the teachers aimed to carry out a more individualised teaching that adapted to the situation and needs of each student, and favoured their participation and motivation (16 people).

*“Cooperative learning, based on projects, or learning based on thinking, individually and adapting ourselves to the needs of each student, ensuring that the lectures were dynamic and similar to what we used to do in the classroom, creating motivating games and activities (p45, MER_ACT_ESAL)”.*

Regarding the participants who taught students with SEN or functional diversity (special education), they carried out *“individualised activities with the help of the counseling and support team (p32, ALUM_NEE)”* (10 participants), through particularly promoting the use of routines. This was completed in order to prevent the students from depending on their families to complete the activities, and to increase their independence in their online lectures, thereby improving their autonomous learning (7 participants).

*“Trying to work routinely in order to increase their autonomy and reduce their dependence on adults (p102, SOLC_DIFC)”.*

Therefore, the differences found between the education levels, with respect to how emergency remote teaching developed, are not entirely significant, and, in any case, they respond to the differences in the pedagogical development carried out in each stage.

#### 3.1.2. Use of Didactic Material and Digital Devices

Regardless of the methodology used, all of the participating teachers required the use of digital devices. Technology is a key element in the development of online lectures during lockdown, such as *“computers, tables and smartphones, as well as cameras for videos and photos (P62, DISPO_DIGT)”*. However, there was flexibility with respect to the type of device demanded by the teachers (96 participants). For instance, it was observed that most of the teachers were flexible about the submission of the activities, adapting the ones implemented to allow the students to complete them using any device that they had at home (72 participants). Usually, the participants made sure that their students had access to a technological resource or device (12 participants). In this sense, some of the teachers requested the students to submit the activities via smartphone, as it is considered as the most common and versatile device, as it has a camera option for videos and photos (43 participants). Nevertheless, some participants also requested that the students use a computer for the completion of their activities (13 participants).

*“Only a computer or a smartphone to send the activities. I am more flexible regarding the submission of the activities, and, sometimes, they send me pictures of their notebooks (p49, DISPO_DIGT)”.*

With respect to the didactic materials used for the lectures, a large number of the participants *“did not create anything, as everything was already done, or took it from the books of the students (p94, CREAR_MAT)”,* and used or adapted other existing materials created by editorials or colleagues, or free-access material on the Internet (62 participants). However, other teachers did create didactic materials of their own, adapted to their methodology and to the needs of their students. On the one hand, some teachers needed to create simple materials, such as explanatory videos, presentations, online stories, etc. (34 participants). On the other hand, other teachers created more advanced didactic resources, such as games, questionnaires, word rolls, etc. (12 participants). Nevertheless, all of the materials created by the participants had exclusive access, and they were never shared with other teachers (40 participants).

*“Yes. Most of my materials are Power Point presentations and videos. I also have pdf documents (p73, CREAR_MAT)”.*

*“I have created a lot of material. I have used tools such as Learning Apps, Live Work Sheet, quizziz, …, (p18, CREAR_MAT)”.*

#### 3.1.3. Follow-Up and Evaluation

The follow-up carried out by the participants was individualised and adapted to the needs of the students, their families and the circumstances of each autonomous community. From the beginning, the teachers did their best to have direct contact with their students (47 participants), *“in different ways, adapting to the possibilities of each of them (p51, CONTAC_ALUMN)”*. This direct contact considered the situation of each student and his/her family regarding their digital resources and knowledge (25 participants), through general communication applications, such as WhatsApp, e-mail and phone calls (21 participants). However, some participants also used the platforms implemented by educational administrations, such as Rayuela in Extremadura (Spain), to inform the families about the activities and communicate with the former in order to perform a follow-up of learning (17 participants).

*“Through e-mail and the platform with have implemented in the school (p72, CONTAC_ALUMN)”.*

Regarding the final evaluation of the students, the teachers stated that it was global and continuous (61 participants), instead of assessing the acquired contents at the end of the subject while *“trying to consider the situation of each student (p13, EVA)”.* From the beginning of the lockdown period, the teachers understood that evaluation could not be conducted through a final exam or test, as the characteristics of the didactic methodology had changed substantially, and the possibilities of performing final tests on the contents were not clear (102 participants). Therefore, some of the teachers carried out the evaluation while taking into account the quantitative mark of the first and second trimester, as well as a qualitative evaluation of the effort and interest of each student in the third trimester (49 participants), in order to safeguard those students who could not access the digital technologies required to follow the lectures.

*“In our case, the third trimester is not going to be evaluated, since not all students have the necessary means. Thus, I will calculate the average of the first two trimesters, and the third trimester will be used to improve that mark, if necessary, or I will value their effort and interest (p131, EVA)”.*

Other participants also pointed out that they adapted to the guidelines created by each autonomous community, *“as was stated by the decree of the department of education (p83, EVA)”* (6 participants).

#### 3.1.4. Adaptation to the New Educational Situation

In general, the adaptation to the new educational situation is defined as a very complex process, due to several aspects (106 participants). Great difficulties were found regarding the adaptation to the hasty new way of teaching online lectures (86 participants).

*“The adaptation of our annual programme in two days to the new situation brought by the COVID-19 pandemic and the need to catch up with educational platforms, applications, websites, videoconference... has posed great stress (P13, DIFIC_PROF)”.*

On the one hand, the teachers stated that this process of adaptation implied excessive work, as they had to modify methodologies, timetables, didactic materials and other elements in order to work remotely (98 participants). All of this had resulted in a moment of labour stress in the teachers (74 participants), causing problems to reconcile their family and professional life in many cases (71 participants).

*“This has required many hours of work. The most difficult aspect was reconciliation and organization at home; I had to sacrifice part of my personal life to attend to the needs of my little students (p66, ADAP_NSE)”.*

In many cases, the workload multiplied, since they had to think about educational alternatives for those families that had problems with technologies. *“Many families lacked materials, interest and knowledge regarding the use of technologies (p25, DIF_PROF)”.* The teachers found it difficult to guarantee that all students had access to the educational contents (29 participants). In this sense, the participants highlighted the lack of digital resources and training of the students and their families (48 participants).

*“The lack of training among teachers, the lack of digital culture of the students and, in some cases, the lack of materials (equipment, access to the Internet, etc.) (p28, DIFIC_PROF)”.*

Other teachers, especially those in early childhood education (17 participants), commented on the difficulty of performing the follow-up with their students (28 participants). The follow-up time is much longer compared to that in face-to-face education (32 participants). In this sense, the teachers claimed that *“the student follow-up was not as effective, due to the lack of affectivity and affection that cannot be transmitted virtually (p81, DIFIC_PROF)”,* and they were distressed by not being able to show the support that children need at such early ages. Moreover, they highlighted the difficulty of accompanying students with SEN, who usually have poorer digital competencies. Thus, they cannot keep the same pace with the rest of their classmates, and they require greater follow-up from their teachers (8 participants). Again, the differences found between the different education levels respond to the characteristics of each stage, thus, overall, there are difficulties with the follow-up of students in early childhood and special education.

*“Making the activities and contents more flexible and adapting them to the different family situations. I believe that the parents will do everything they can, since these are their children, but they are not all in the same level. I have tried to send clear activities, with videos accompanied by my explanations, which children aged 7/8 years could perform somewhat autonomously (p51, ADAP_NSE)”.*

In some cases, this situation worsened due to the initial lack of digital competencies among the teachers (47 participants), which caused a serious problem when adapting in such a hasty manner to remote teaching; for instance, some of the teachers *“had to self-learn about new educational technologies and applications hurriedly (p29, ADAP_NSE)”* (32 participants).

In this situation, the role of collaboration and companionship among teachers was a very important aspect (28 participants), in order to face the deficiencies in digital competencies. One of the most common ways to learn the use of digital applications, which ensured the capability to perform them adequately in the online classroom, was to ask colleagues in the centre for help and test such applications before implementing them (13 participants).

*“Aprendiendo deprisa y poniendo en práctica lo aprendido, primero “probando” con los profes para ver si era viable (p77, SOLC_DIFC)”.*

However, other participants stated that their adaptation to the new situation of teaching through ICT was not as difficult, since their centres already worked with them and had participated in innovation projects. Therefore, this change was not too abrupt for them (36 participants), demonstrating the importance of acquiring teaching digital competencies, in order to face any educational emergency situation in a more positive manner.

*“Quite well. In my centre, we are immersed in an educational innovation project with new technologies. Since we already used a digital platform to upload activities and theoretical content for the students, the change did not pose great difficulties (p57, ADAP_NSE)”.*

### 3.2. Socio-Digital Inequalities of the Students and Family Accompaniment for Learning

#### 3.2.1. Follow-Up and Support by the Families

In a general manner, the families tried to provide the necessary support to the students for the development of their learning at home. The teachers pointed out that their students received optimal support from their families (56 participants); in fact, they stated that the families *“were participatory and interested in the evolution and learning of their children (p6, APOY_FAM)”.* However, another group of teachers claimed that the families provided the same support with respect to face-to-face education; that is, they did not see any extra support from the families (42 participants) and highlighted that *“there had been no further collaboration from the families compared to the situation before the state of alert (p10, APOY_FAM)”*.

In any case, the teachers mentioned that the support and follow-up of the families regarding their children was within their capabilities (40 participants) and *“did it as they could. Many of them worked from home and, sometimes, they could not provide the adequate help (p18, APOY_FAM)”*. In this sense, one of the most remarkable factors was the amount of time that the families had, since many of them had to work both inside and outside of the home and reconcile their job with other duties. This made it difficult to have enough time to assume the necessary support required by the students (37 participants).

*“The personal circumstances of each family vary a lot. Some families had to work, take care of their children, do their chores... and they did not have the same availability as others (p81, DIF_ORIFA)”.*

Some teachers pointed out that having older siblings was a protective factor and reduced the difficulties of the families in the support of learning, since the students who had older siblings received greater help in comparison to those who did not have older siblings (3 participants).

*“Those who have older siblings had it easier, as they already know the subject matter. They also operate digital devices more easily (p18, DIF_ORIFA)”.*

#### 3.2.2. Learning Patterns and Routines

Regarding the learning patterns and routines established in each household for the study, *“in most cases there was no pattern (p134, PATR_APREN)”* determined by the parents (65 participants), which, in many cases, especially in children with SEN, did not allow the students to follow the pace of the class, due to the lack of knowledge of the parents to help and give them guidelines (11 participants).

*“I teach Hearing and Speech. All my students have educational needs. In some cases, it was impossible to continue with the teaching-learning process, since the parents did not have the necessary knowledge to work with their children (p34, ALUMN_NEE)”.*

In this context, most of the participants stated that the teachers themselves were the ones who gave the guidelines through assignment deadlines or motivation (27 participants). Some teachers even developed a document with steps or a routine to follow at home, so that the families knew what to do with their children (4 participants).

*“They were given a set of instructions that the tutors unified in a document that we shared in Drive for the families, and then each teacher informed his/her students (p18, PATRO_APREN)”.*

However, there were also families that established routines, as they understood that setting times and activities throughout the day were very important for the emotional development and learning of their children (35 participants). Usually, these were families who were already involved in the education of their children, and had a higher education level.

*“The routine chosen by the family. The emotional support of the child is more important than the contents of the subject (p130, PATR_APREN)”.*

According to the interviewed teachers, the students of these committed families, who established learning patterns and routines for their children, obtained a higher academic performance (42 participants). It can be asserted that the education level and the participation in the centre are directly related to the support provided to the students, and, therefore, to their academic performance and success.

*“The more committed families are in fact the ones whose children are more advanced in class (p102, APYO_LOGESC)”.*

#### 3.2.3. Situation of Socio-Digital Inequalities of the Students

Due to the health crisis situation in which we have lived, it has been demonstrated that there are obvious socio-digital inequalities among students. The participants did not find a single student in urban centres and medium or high socio-economic situation without the necessary digital resources, or who did not have the digital competencies required to follow the daily lectures and proposed activities (33 participants). Some teachers even stated that, in general, there was no digital divide and that *“the children did not have any problem with technologies (p91, ALUM_SINCPDG)”.*

However, some participants pointed out that *“over half of the families did not have digital resources (p19, ALUM_SINCPDG)”*, especially among students of a low socio-economic level. These students did not have the necessary resources and digital competencies to follow the pace of the online lectures, thus, some of them could not complete the academic course adequately (27 participants).

*“The problems were those already mentioned, that is, connection problems, malfunctioning computers, or parents and students who did not have the necessary competences to follow the lectures (p15, ALUM_SINCPDG)”.*

They also highlighted the issues related to the Internet connection in rural areas, which made it difficult for the teachers to share the digital materials they created and the activities that they proposed (21 participants).

*“In rural areas, even today there are no means to connect to the Internet, so it is impossible to share the materials and proposed activities (p31, ALUMMN_SINRCD)”.*

#### 3.2.4. Socio-Digital Inequalities among Families

Other factors that showed differences in the support and follow-up of the families in the learning of students were the socio-economic level and the urban/rural context of the educational centre, which influenced the access to technologies and the digital competencies of the parents, respectively. In this way, those families with lower socio-economic (25 participants) and education (17 participants) levels had more difficulties in helping and accompanying their children in their learning.

*“The causes are the lack of studies, different socio-economic levels and, above all, the lack of time and reconciliation with the professional life (p125, DIF_ORIFA)”.*

The socio-economic level influences the differences found among families, especially in *“the access to technology (p61, DIF_ORIFA)”*. Thus, in their digital competencies, this further reduces the possibility of family support for the students (16 participants) in the realisation of the digital activities.

*“Some families provided guidance to their children because they had the time and the digital competences required; however, instead of helping, other families needed their children to teach them, as they did not have such digital competences (p75, DIF_ORIFA)”.*

With respect to the context of the educational centre, the teachers in urban centres pointed out that the families of their students had *“medium-high digital competences, except for a few cases (p45, CMP_DIG) (57 participants)”* and, therefore, they had no additional difficulties in accompanying their children in the realisation of the digital activities. On the other hand, most of the teachers from rural centres (25 participants) found that families had low or very low digital competencies.

*“Not everyone can access the activities, as they do not have the necessary digital competences. They know how to use their smartphones very well, but they do not know how to use the platforms properly (p98, CMP_DIG)”.*

In terms of the type of educational centre, there were no significant differences between public and charter centres. The teachers from charter centres highlighted that they worked with families *“of the entire spectrum, that is, from great to null preparation (p76, CMP_DIG)”* (24 participants). The teachers from public centres stated that the families that they worked with showed *“medium-level digital competences (P12, CMP_DIG)”*, in general (45 participants).

#### 3.2.5. Solutions Developed for Students in a Situation of Socio-Digital Inequality

In these contexts, the work of the educational institutions, as well as the measures implemented by the local and regional governments, were very helpful in the promotion of technological access and Internet connection, since, in many cases, such materials and resources were provided (34 participants). In rural areas, *“the social services and the city hall provided the necessary material (p2, ALUM_SINRCD)”*, and became the main agents in the promotion of equality (18 participants). However, in rural areas, *“the school provided electronic devices to families with less resources (p126, SOLC_DIFC)”*; it was mainly the educational centres who made the effort to ensure a more equitable education (21 participants). Nevertheless, other teachers (7 participants) mentioned that this was a collaboration between institutions, especially between the educational centres and the regional administrations.

*“Yes, but the centre provided them with laptops so that they could access the activities. The government also facilitated Internet connection when necessary (p42, ALUMN_SINRCD)”.*

Lastly, only a minority of teachers commented that there were no solutions to the problem of access to technology among students, and, when such solutions were implemented, it was too late (5 participants). Therefore, these teachers approached their teaching with such difficulties.

*“To date, I believe that they do not have the economic means to connect to the Internet or access the materials and activities. We did not find any solution to their problems (p74, ALUMN_SINRCD)”.*

Nevertheless, regardless of whether the connectivity problems were solved or not, the teachers had to overcome these difficulties, especially at the beginning, when the main problems were detected. On the one hand, a group of teachers solved such problems by contacting the families directly via phone calls or mobile applications (17 participants).

*“Yes, I actually sent them the activities in pdf also through WhatsApp (p1, ALUM_SINRCD)”.*

Another group of teachers adapted the activities to the needs of each student, even transforming them to fit their competence level and situation, thus, facilitate their access (26 participants).

*“Yes, adapting myself to their situation, with activities adapted to their competence level (p21, ALUM_SINCPDIG)”.*

In this entire process, it was highlighted that *“fortunately, the families helped a lot (p32, ALUM_SINCPDIG)”* to solve the problems related to technological access, showing great interest and helping their children as much as they could (5 participants).

#### 3.2.6. Preventing the Digital Divide in Future Closing of Educational Centres

To prevent these inequalities in an educational future in which ERE is developed, different measures and strategies are proposed to favour technological access, Internet connection and an adequate development of digital competencies in all students (51 participants). On the one hand, it is necessary to establish social support measures, providing devices for both families with little resources and the educational centres (14 participants).

*“Support to ensure that all students have some device with which they can access the lecture. Access to the Internet through some help or plan; this is not about the future, but the present (p65, IDE_NODES)”.*

Moreover, some teachers propose that ICT be implemented from the first stages of education, to ensure that students have the necessary digital competencies from the educational centres, in order to compensate for the lack of technological resources and skills in the use of such devices among students (6 participants).

*“Implementing ICT from the first years of school, although this is already being done in many centres (p136, IDE_NODES)”.*

The above mentioned requires “further investment in education and training in ICT for students and teachers (p111_IDE_NODES)”; that is, it is necessary to purchase digital devices for students and centres, and teachers must be trained in ICT competencies and emergent methodologies in order to be capable of solving the problems derived from the digital divide, typical of ERE (27 participants).

*“Further training for teachers in this area and more technological materials for the classrooms (p66, IDE_NODES)”.*

Therefore, in a pandemic situation, it becomes clear that the participation of the families is very important for the education of their children, the follow-up of their activities and the establishment of learning patterns and routines. This requires overcoming the difficulties related to socio-economic and educational level, access to technologies and digital competencies, as well as helping the families to carry out an adequate daily follow-up of their children.

*“It is fundamental that the families collaborate in this situation, as it is very difficult to carry out a daily follow-up of each student in this situation of remote education (p74, APYO_LOGESC)”.*

## 4. Discussion

### 4.1. What Methodological Changes Were Made to Adapt to ERE?

The closing of educational centres due to the COVID-19 pandemic has been a disruptive process, with great challenges to all education systems around the world [14]. It has provided the opportunity to identify other ways of teaching and incorporate technologies in teaching practice in a real manner [17]. However, although a significant educational change has occurred, in which technologies are the key element of ERE, there are differences in the ways of adapting or developing it [4]. The traditional model of content transmission predominates over the use of active and emergent methodologies, maintaining the unidirectional idea of sharing documents or audio-visual content [8]. As has already been pointed out by other studies, it seems that the teaching model implemented by teachers depends on the model developed before the lockdown situation [8].

However, in view of the obtained results, it can be asserted that ERE is characterised by: (1) the flexibility of teachers in the realisation and submission of the activities, in order to adapt to the needs and capacities of the students; (2) the use of digital didactic materials to ensure the transmission of content; (3) the importance of carrying out an individualised and continuous follow-up of the students regarding ICT; and (4) the realisation of a global and continuous evaluation process that assesses aspects, such as motivation for and participation in learning, without objective tests on the content.

### 4.2. What Challenges Did Teachers and the Educational Community Have to Face during the Lockdown?

In general, we highlight that most of the teachers approached this new and sudden educational situation in a very positive manner. Some teachers showed great adaptability in responding to the challenging COVID-19 situation [18]. However, the process was not exempt from difficulties, as it was mainly characterised by the work stress experienced by most of the participants. It is important to take into account that the teachers had to modify their teaching method to an online format in a very short time, alongside the problems of reconciliation with family life and the lack of teaching digital competencies in a significant proportion of teachers [10,11,13,15]. Moreover, they also had to face problems in the scope of the student’s family as well as the specific needs of each student, and solve such problems in the most efficient way possible.

In this context, the importance of collaboration among teachers as well as participation in innovative projects related to the introduction of technologies in educational centres became obvious, as these factors helped teachers to adapt to ERE in a less stressful manner. Teachers are recommended to be trained in online learning [6].

### 4.3. How Did Socio-Digital Inequalities Manifest throughout the Lockdown?

The closing of educational centres revealed that the digital divide exists among students from low socio-economic level and/or rural contexts [5,6,16,34], since these two factors are mainly characterised by the lack of resources and connection to the Internet, which makes it difficult for these students to follow the course adequately [5,10,13,17,18,29,35,36]. As was reported by previous studies, it is important to guarantee that all students have access to technology and connection to the Internet, in order to improve the teaching–learning process during a time of crisis, such as the COVID-19 pandemic [6]. Thus, both the educational institutions and the local and regional governments have provided students with technological devices and Internet connection [5,8], although, in some cases, such support did not arrive in time or was not enough. However, the digital competencies of the families are related to the academic success of the students, since such competencies are necessary to accompany the students in the development of their activities with ICT. Therefore, in order to prevent socio-digital inequalities in the future, apart from providing and lending technological resources and Internet connection, one of the most important solutions is the digital training of both families and students [6].

### 4.4. How Did the Families Approach the Participation and Accompaniment of the Learning of Their Children?

One of the main agents in ERE is the student’s family, since, along with the teacher, they accompany and support the learning of the students, with the aim of helping the latter to achieve their academic success [18,34,36]. In this sense, two types of families were found, which approached this situation in a very different manner. On the one hand, the families that belonged to a medium-high socio-economic level, with a higher education level and basic acquired digital competencies, were characterised as providing support and accompaniment in the learning and structure of learning routines; the students of these families did not find significant difficulties in following the online lectures. On the other hand, the families with a low socio-economic level who lived in rural areas, with a basic education level and without digital competencies in many cases, were characterised as not having enough knowledge to provide the necessary support for their children, and they depended on the learning routine organisation given by the teachers; this made it difficult for these students to follow the virtual classes, thus finding themselves in a situation of vulnerability. This scenario demonstrates the lack of training among families in matters of remote education, especially among families with a lower education level and limited resources [35]. Thus, it can be asserted that during a lockdown period, the academic results of the students depend, to an even greater extent, on the education and digital competencies of their families to accompany their learning [16,18,36,39].

The consequences of the educational lockdown urge the promotion of measures and solutions, which should also be sensitised with those particular students in a greater situation of social disadvantage [39].

### 4.5. Limitations and Future Research

The present study was carried out from a holistic approach of the phenomenon of socio-digital inequalities in education with respect to the COVID-19 pandemic, with the aim of considering all of the possible variables and aspects that may influence said phenomenon. However, it must be pointed out that the approach of the study in these terms was very ambitious, due to its complexity and amplitude. On the one hand, each of the questions considered in the study can pose a study object on their own, which, if analysed separately, may be studied more thoroughly. Moreover, the amplitude and variety of the sample, along with the thorough and rigorous analysis process, allowed highly reliable results to be obtained and the visualisation of the real situation in a precise manner; however, as in any qualitative study, there are biases related to the need of the interviewee to satisfy the interviewer in the answers to the questions, as well as biases regarding the interpretation of the data. Therefore, it is necessary to compare the results with those of quantitative studies that have had the same study object, and replicate some of these in this context.

## 5. Conclusions

Understanding the research process from a cyclical conception, we conclude that there remains numerous questions unanswered, thus, opening new research lines for future studies to expand and improve the data obtained in the present study, which was focused on the socio-digital inequalities in education regarding the COVID-19 lockdown.

However, it can be asserted that ERE has proved very useful during the closing of educational centres, demonstrating its potential to transform education [7,14,17]. Nevertheless, it was also shown that the development of an ERE model can cause socio-digital inequalities among students, due to the lack of access to digital devices and Internet connection. This is mainly influenced by factors, such as the socio-educational level of the family and the rural/urban context of the educational centre [35].

Therefore, it is necessary to provide technological resources to both educational centres and families with low resources. In this sense, the policies of educational centres and local and regional administrations were very useful in the provision of all types of digital devices to those students who needed them. Moreover, it is essential to provide teachers with training in teaching digital competencies, in order to develop ERE models based on more active methodologies [6], as well as to teach digital and educational capacities to the families for the correct accompaniment of their children.

It is necessary to delve into the possible effects of the digital divide and how it evolves over time, as well as its factors and consequences, in order to adapt as much as possible to the policies and strategies to ensure that the educational-digital environment proposed is ideal for everyone [28,39]. The strategy must have an integral approach that addresses all the realities of the students [6]. The findings of this study are expected to help in the identification and reduction of the digital divide, and in the prevention of future problems associated with it.

## Figures and Tables

**Table 1:** System of emergent dimensions, categories and codes.

Dimension	Category	Code
Practice Teacher	Way of adapting to the new educational situation	ADAP_NSE
	Way of contacting the students	CONTAC_ALUM
	Teaching methodologies and activities carried out during the state of alert	MET_ACT_ESAL
	Digital devices were necessary to carry out the methodologies and activities	DISP_DIGT
	Characteristics of the didactic materials used	CREAR_MAT
	Adaptation of the student evaluation	EVA
	Difficulties found by the students	DIFIC_PROF
	Solutions to difficulties	SOLUC_DIFC
Family support and accompaniment	Socio-educational level of the family	NVEL_FAM
	Type of digital competencies of the families	COMP_DIG
	Learning patterns of the students	PATR_APREN
	Support and orientation of the families	APOY_FAM
	Support differences between families	DIF_ORIFA
	Consequences of family support in academic achievements	APYO_LOGESC
Socio-cultural inequalities	Students with/without digital resources and access to the Internet, and problems tackled	ALUM_SINRCD
	Students with/without digital competencies and problems tackled	ALUM_SINCPDG
	Students with special educational needs and problems tackled	ALUM_NEE
	Other problems related to the digital divide	OTR_PROB
	Prevention of inequalities and the future of education	IDE_NODES

## Data Availability

The data presented in this study are available on request from the corresponding author.

## Appendix A

**Data of the Google Form questionnaire:**

- Autonomous Community to which the centre belongs.
- Educational stage in which you teach (early childhood education, primary education, secondary education, special educational needs or hearing and speech).
- Socio-cultural situation of the area of the centre (low, medium, high).
- Educational context (rural/urban).
- Type of centre (Public, Private, Charter).

**Protocol of the semi-structured interview:**

***(A) Teaching methodology:***

- How did you adapt to the new educational situation?
- How did you keep in contact with your students?
- What types of teaching methodologies and activities did you carry out during the state of alert?
- What digital devices did you need to perform these?
- Did you create digital didactic materials? What are their characteristics? Could you share the link if they are open-access?
- How will you approach the evaluation?
- What type of difficulties did you find as teachers in this change in the way of teaching?
- How were these difficulties solved?

***(B) Information about the family of the students***

- In general, what was the education level of the families of your students?
- What type of digital competencies did the families have to support and guide the students at home?
- What were the learning patterns of the students at home?
- How did the families provide support and guidance for the learning of the students at home?
- What type of differences did you detect in the support and guidance among families? What do you think was the main cause?
- How are the learning patterns of the students and the type of support of the families related to academic success?

***(C) Information about the digital divide and inequalities***

- Did you have any case of a student who did not have the necessary digital devices? How was this difficulty tackled?
- Did you have any student who did not have the necessary digital competencies to carry out the activities? How was this difficulty tackled?
- Did you have any student with SEN or functional diversity? How was this difficulty tackled?
- What other types of problems were encountered in following the teaching methodology established? How was this tackled?
- What ideas would you propose to prevent inequalities in the future regarding education and ICT?
